# The development and validation of the major life changing decision profile (MLCDP)

**DOI:** 10.1186/1477-7525-11-78

**Published:** 2013-05-08

**Authors:** Zaheer U Bhatti, Sam S Salek, Charlotte E Bolton, Lindsay George, Julian P Halcox, Sharon M Jones, Ian R Ketchell, Richard H Moore, Ramsey Sabit, Vincent Piguet, Andrew Y Finlay

**Affiliations:** 1 Centre for Socioeconomic Research, Cardiff School of Pharmacy and Pharmaceutical Sciences, Cardiff University, Redwood Building, King Edward VII Avenue, Cardiff CF10 3NB, UK; 2 Department of Dermatology and Wound Healing, Glamorgan House, Cardiff University School of Medicine, Heath Park, Cardiff CF14 4XN, UK; 3 Department of Respiratory Medicine, Cardiff University School of Medicine, University Hospital of Wales, Heath Park, Cardiff CF14 4XW, UK; 4 Current address: Nottingham Respiratory Research Unit, University of Nottingham, City Hospital, Clinical Sciences Building, Hucknall Road, Nottingham NG5 1PB, UK; 5 Department of Diabetes and Endocrinology, University Hospital Llandough, Penlan Road, Penarth, Vale of Glamorgan CF64 2XX, UK; 6 Welsh Heart Research Institute, Cardiff University School of Medicine, Heath Park, Cardiff CF14 4XW, UK; 7 Department of Rheumatology, University Hospital of Wales, Heath Park, Cardiff CF14 4XW, UK; 8 Adult Cystic Fibrosis Services, University Hospital Llandough, Penlan Road, Penarth, Vale of Glamorgan, CF64 2XX, UK; 9 Department of Nephrology and Transplantation, University Hospital of Wales, Heath Park, Cardiff CF14 4XW, UK; 10 Department of Respiratory Medicine, University Hospital Llandough, Penlan Road, Penarth, Vale of Glamorgan CF64 2XX, UK

**Keywords:** Major life changing decisions, MLCD, Major life changing decision profile, MLCDP, Chronic disease impact, Long-term impact, Life events, Life course, Life decisions, Quality of life, Psychometric properties

## Abstract

**Background:**

Chronic diseases may influence patients taking major life changing decisions (MLCDs) concerning for example education, career, relationships, having children and retirement. A validated measure is needed to evaluate the impact of chronic diseases on MLCDs, improving assessment of their life-long burden. The aims of this study were to develop a validated questionnaire, the “Major Life Changing Decision Profile” (MLCDP) and to evaluate its psychometric properties.

**Methods:**

50 interviews with dermatology patients and 258 questionnaires, completed by cardiology, rheumatology, nephrology, diabetes and respiratory disorder patients, were analysed for qualitative data using Nvivo8 software. Content validation was carried out by a panel of experts. The first version of the MLCDP was completed by 210 patients and an iterative process of multiple Exploratory Factor Analyses and item prevalence was used to guide item reduction. Face validity and practicability was assessed by patients.

**Results:**

48 MLCDs were selected from analysis of the transcripts and questionnaires for the first version of the MLCDP, and reduced to 45 by combination of similar themes. There was a high intraclass correlation coefficient (0.7) between the 13 members of the content validation panel. Four more items were deleted leaving a 41-item MLCDP that was completed by 210 patients. The most frequently recorded MLCDs were decisions to change eating habits (71.4%), to change smoking/drinking alcohol habits (58.5%) and not to travel or go for holidays abroad (50.9%).

Factor analysis suggested item number reduction from 41 to 34, to 29, then 23 items. However after taking into account item prevalence data as well as factor analysis results, 32 items were retained. The 32-item MLCDP has five domains education (3 items), job/career (9), family/relationships (5), social (10) and physical (5). The MLCDP score is expressed as the absolute number of decisions that have been affected.

**Conclusions:**

The 32-item (5 domains) MLCDP has been developed as an easy to complete generic tool for use in clinical practice and for quality of life and epidemiological research. Further validation is required.

## Background

The focus of health-related quality of life (HRQoL) research has been on the assessment of current disease impact on patients’ lives. However disease may have an additional major long-term impact by influencing major life changing decisions (MLCDs) [1,2], such as those concerning education, career, relationships or having children [3-7].

There is little information available about the impact of chronic diseases on MLCDs and there has been no defined measure to capture this information [1]. There are many published surveys assessing the impacts of chronic diseases on patients’ HRQoL. However, these studies have not addressed the long term impact of chronic disease on critical life decisions taken by patients [1]. Instruments to measure HRQoL usually assess patient’s current experiences and are not designed to assess the long term impact of disease, which may change over time. Even follow up studies, which might be expected to encompass more long term issues, usually compare current impacts before and after. In our review of HRQoL instruments we could not identify any instrument containing items to capture the influence of chronic disease on MLCDs or any long term impact on patients’ lives [1].

A method to measure this impact is needed to draw attention to the issues, to identify specific patient needs, to allow a more complete assessment of the burden of disease and to permit comparison between the impacts of different diseases; with timely identification of the needs of patients, appropriate intervention may enable patients with chronic conditions to fulfil their full potential and aspirations. The aim of this study was to develop a standardised tool for use across all chronic conditions to record the influence of chronic disease on MLCDs and initially validate such a measure.

## Methods

The South East Wales Research Ethics Committee approved this cross-sectional prospective study on 2nd June 2008. All participants gave informed written consent.

### Study participants

Patients (aged 16 years and above) who had had chronic disease for > 1 year were recruited from the outpatient departments of dermatology, cardiology, rheumatology, nephrology, diabetes and respiratory disorders at the University Hospital of Wales, Cardiff and University Hospital Llandough. Patients were excluded if suffering from other illness or disability.

### Data processing and analysis

Data was processed using SPSS16 statistical software for Windows. Descriptive statistics were used to record the judges' responses (%) on a four point scale. Statistically, Kappa coefficient and intraclass correlation coefficient (ICC) techniques were used to evaluate the agreement among the panel of judges for inter-rater reliability. For non-quantitative data (categorical/nominal), the kappa coefficient is the technique of choice. The ICC is more appropriate and better than the kappa technique for analysing data obtained from ratings using any scale (e.g. from 1 to 10) [8,9].

The level of agreement among the panel members for each item of the MLCDP was assessed statistically by using ICC for the quantitative and kappa technique for the qualitative phase to measure the level of agreement (inter-rater reliability) among the panel of judges. The SPSS software generally provides kappa statistics for only two judges. The Cohen Kappa macro syntax file was computed using SPSS12 software to calculate the multiple inter-rater agreement (>2 raters).

### Procedure

There were four stages; conceptualisation and item generation, item reduction and development of the first version of a new tool, content validity and development of a revised version and construct validation using factor analysis. Using a formal check list [10], we conceptualised the development of a self-administered general health profile with multi-dichotomous close-ended simple statements.

Data for item generation were collected from a postal survey and individual interviews. Core themes and categories were identified using content analysis [3-7], and a generic MLCD questionnaire was developed, the “Major Life Changing Decisions Profile” (MLCDP). NVivo 8 and SPSS16 software were used.

The items generated were analysed using standard qualitative strategies [10-12] to reduce them to the core items identified from the interview transcripts. Some population and gender specific items were retained. After qualitative analysis, taxonomy techniques and rephrasing of items/statements were introduced to fit the items into categories. Standard methods [10] to assess and modify language, reading age and item lengths were applied.

The 45-item MLCDP (version 1) was content validated by a team of experts (panel of judges). Their task was to review the profile, rate each item and to suggest appropriate changes to develop a profile that could accurately measure what it was intended to measure, with appropriate focus and emphasis for the target population. Consultant physicians, specialist nurses and academic experts from different disciplines were invited to join the panel of judges and to take part in this process. The content validation process was carried out in two phases, quantitative assessment and qualitative assessment.

For quantitative assessment, the MLCDP (version 1) and the questionnaire items rating sheets were sent out to the panel of judges to rate each item and for their expert opinion. The members of the panel were asked to bring their items rating sheets for discussion at the subsequent meeting. Instruction was provided for rating of the MLCDP (version 1) on a four point Likert type ordinal scale (1 = Strongly agree, 2 = Agree, 3 = Disagree and 4 = Strongly disagree) for each of the four criteria of language clarity, completeness, scaling and relevance. Under each item, a separate section was provided for the judges to write any suggestions.

In the qualitative assessment stage, all 13 panel members returned their completed item rating sheets and 6 panel members attended the discussion. The discussion was digitally recorded for later analysis. During the meeting, patients’ comments given at the conceptualisation stage were consulted again where necessary to clarify the discussion. Various changes were made with consensus to the questionnaire (item inclusion, retraction, modification, and sequence) to reduce ambiguity and to make the MLCDP simpler, more user friendly and easier to understand.

This revised MLCDP was administered to another cohort of subjects for further construct validation using the Exploratory Factor Analysis technique of “principle component analysis” [13]. A correlation and component matrix was created for the assessment of data suitability followed by application of the Kaiser-Meyer-Olkin (KMO) measure and Bartlett’s test of sphericity for adequate sampling. For factor extraction, Kaiser’s criterion and Cattell’s scree test were carried out and the varimax technique was used for factor rotation and final interpretation. Cronbach’s alpha statistics were used to measure internal consistency [14].

Face validity and practicality of the new tool was assessed by using a separate simple tick box questionnaire to record patients’ views on the new tool and by measuring time required to complete the MLCDP.

## Results

655 patients were invited to take part, of whom 365 (55.7%) responded. There were 308 (83.7%) evaluable responses (postal survey = 258, individual interviews = 50). The disease duration of these subjects ranged from 2–61 years (median = 18). The frequency and percentage of the emerged MLCD themes have been previously reported [3-7].

### Domain generation

15 main MLCD categories and some individual items were identified (3–7) and initially grouped under six MLCD domains, namely: “education”, “career/work”, “family/relationships”, “social”, “physical” and “major treatment decisions” (Table 1).

**Table 1:** Initial MLCD domains with main categories of decision types


A. Education	
	Education
B. Career/Work	
	Career choice
	Early retirement
	Job
	Professional bodies
	Professional sports
C. Family/Relationships	
	Having children
	Relationships
D. Social	
	Lifestyle (smoking, drinking alcohol)
	Holidays/travel abroad
	Housing
	Move abroad
	Move city
	Clothing
	Swimming
	Not socialise
	Make up
E. Physical	
	Sports
	Driving
F. Major treatment decisions	
	Dialysis
	Organ transplant
	Surgery

### Item/Statement generation

The survey responses and interview transcriptions were cross-referenced during the decision-making process of using the themes for construction generation of each item. Items were kept short and phrased in the past tense. The core MLCD domains and statements were arranged sequentially to reflect life stages.

Two non-MLCD “life style” issues “to take part in other sports activities” and “to give up driving” were grouped under the “physical” domain. The items related to “community activities” and to “wear a wig/toupee” were included in the “social” domain. The gender specific MLCD “wearing make up” was addressed separately.

### Item reduction

Initially, 48 items/statements were generated. Statements considered inappropriate, ambiguous or not universal were deleted. Several items were rephrased or combined. Items mentioned by less than 5% of respondents were not included.

The theme “not to mention illness on job application” was removed as this was considered an inappropriate action. The theme “not to seek employment” was removed because of similarity with another theme. One independent main category “to quit from professional sports” was excluded as it was reported by <5% of the patients. However some gender and speciality specific categories were retained, such as “major treatment decisions” and “to wear make up”. The statement, “To leave a professional association/committee” was retained because of its generic nature. One life style MLCD related to “swimming” was put under the “social” rather than the “physical” domain: it was only reported by patients who were suffering from skin conditions, but this item may also be of relevance to patients with physical conditions. Another item “I decided not to take part in other sports activities” was added to the “physical” domain.

### Development of the draft MLCDP

The resulting 45-item draft profile covered six MLCD domains related to education (4 items), career/work (13 items), family/relationships (8 items), social (16 items), physical (3 items) and major treatment decisions (1 item). A 4-point (5 response options) unipolar “adjectival” scale (No influence = 0, Slight influence = 1, Moderate influence = 2, Strong influence = 3 and A very strong influence = 4) was initially adopted for the MLCDP [10].

In the designing of the MLCDP we decided that we wished to measure the total impact of all of a patient’s health conditions rather than focus on the effect of one specific disease. The reason for this is that if a patient has more than one condition affecting MLCDs, then it is highly likely that both conditions would influence in different additive ways these decisions and it would often be artificial to ask a patient to try to specify which MLCD was influenced by a specific disease.

### Psychometric evaluation: content validity

The 45-item MLCDP was presented to a panel of experts for content validation [10,15]. The 13 panel members included clinicians from seven participating medical specialities: seven consultant physicians (5 males, 2 females) and six specialist nurses (all female). Eleven panel members were independent from the research team. Each panel member rated each of the 45 items on a 4-point scale (strongly agree to strongly disagree) for each of the four criteria, language clarity, completeness, scaling and relevance and suggested changes where they identified issues with any of these criteria. For each criterion, the percentage of panel members that strongly agreed, agreed, disagreed and strongly disagreed with that criterion having been fulfilled was calculated (Table 2).

**Table 2:** Responses from the expert panel

**Response option**	**Frequency of panel members’ ratings across four criteria of the 45 items (%)**			
	**Language clarity**	**Relevance**	**Completeness**	**Scaling**
Strongly agree	58	55	50	57
Agree	28	33	33	28
Disagree	12	9	13	13
Strongly disagree	2	3	4	2

For each criterion the percentage is given of the 13 panel members agreeing with that criterion being fulfilled.

The median values of the panel members’ ratings indicated good agreement between them in all four criteria (Table 2). The items that did not gain agreement across all four criteria were “I decided not to take promotion”, “I decided to stay in the same employment”, “I decided to leave a professional association/committee” and “I decided not to go swimming”. The following items failed on three criteria: “I decided to change my choice of career”, “I decided to give up a job”, “I decided to have IVF (In Vitro Fertilisation) treatment”, and “I decided to change my eating habits”.

The Intraclass Correlation Coefficient (ICC) analysis of absolute agreement showed an ICC of 0.707 (p = <0.0001; CI = 0.61 to 0.78) [8] indicating good inter-rater reliability among the panel and supporting the content validation.

Four clinicians (2 males, 2 females) and two academic QoL experts (both male), took part in the subsequent qualitative panel discussion. Each item was discussed until consensus was reached on their retraction, addition or modifications. The panel members’ responses had a Cohen’s Kappa [8] value for multiple raters (n = 6) of 0.81, (p = <0.0001, CI = 0.69 to 0.93). This value indicates “almost perfect agreement” [16] among the panel members on the 45 items, further supporting the content validity of the MLCDP.

Changes were implemented to produce the 41-item MLCDP. The six MLCD domains were reduced to five: education (4 items), job/career (10 items), family/relationships (7 items), social (15 items) and physical (5 items). This revised version was then examined by factor analysis.

### Factor analysis

225 patients were invited by post to complete the 41-item MLCDP: 15(6.6%) declined. 210 (93.3%) patients (30 from each of the seven specialities, with 32 different chronic conditions, mean disease duration = 19 yrs (range 2–74) (Tables 3 and 4) completed the 41-item MLCDP. The MLCDP was printed on thick blue paper to increase the response rate [17]. All 210 patients answered all of the items.

**Table 3:** Socio-demographic characteristics of the study participants (n = 210)

**Variables**	**Number (%)**
**Age (years)**	
Minimum-Maximum	16-89
Median	52
Mean	50.8
**Gender**	
Female	108 (51.4)
Male	102 (48.6)
**Education**	
School	122 (58.1)
College	52 (24.8)
University	36 (17.1)
**Marital status**	
Single/Divorced/Widowed	98 (46.6)
Married/Living with partner/Civil partnership	112 (53.4)
**Employment status**	
Employed/Self employed	77 (36.7)
Unemployed	42 (20)
Retired/Early retirement	88 (41.9)
Housewife/Student	3 (1.4)

**Table 4:** Prevalence of different disease states in the study participants (n = 210)

**Specialities/diseases**	**N**	**Percent**
**1. Cardiology**		
Coronary artery disease	7	3.3
Congenital heart disease	12	5.7
Atrial fibrillation	6	2.9
Myocardial infarction	3	1.4
Congestive pericarditis	1	0.5
Congestive heart failure	1	0.5
**2. Nephrology**		
Chronic Kidney Disease IV	23	11.0
Chronic Kidney Disease V	7	3.3
**3. Respiratory Medicine**		
Chronic Obstructive Pulmonary Disease	30	14.3
**4. Cystic Fibrosis**	30	14.3
**5. Diabetes**		
Diabetes Type 2	23	11.0
Diabetes Type 1	7	3.3
**6. Rheumatology**		
Rheumatoid arthritis	12	5.7
Ankylosing spondylitis	1	0.5
Psoriatic arthritis	6	2.9
Osteoarthritis	1	0.5
Polymyalgia rheumatica	1	0.5
Systemic lupus erythematosus	2	1.0
Sjorgen’s syndrome	1	0.5
Reflex sympathetic dystrophy	1	0.5
Connective tissue disorder	1	0.5
Sarcoidosis	1	0.5
Antiphospholipid syndrome	1	0.5
Fibromyalgia	1	0.5
Reflex sympathetic dystrophy	1	0.5
**7. Dermatology**		
Psoriasis	16	7.6
Atopic eczema	8	3.8
Acne	2	1.0
Hidradenitis suppurativa	1	0.5
Alopecia areata	1	0.5
Pityriasis lichenoides chronica	1	0.5
Behcet’s syndrome	1	0.5
Total	210	100.0

The internal consistency of the 41-item MLCDP measured by Cronbach’s alpha was 0.84, indicating good reliability [18]. Seven items had corrected total-item correlations of <0.2 and were removed, increasing the Cronbach’s alpha of the 34-item MLCDP to 0.85 (Appendix 1).

**Figure 1:**
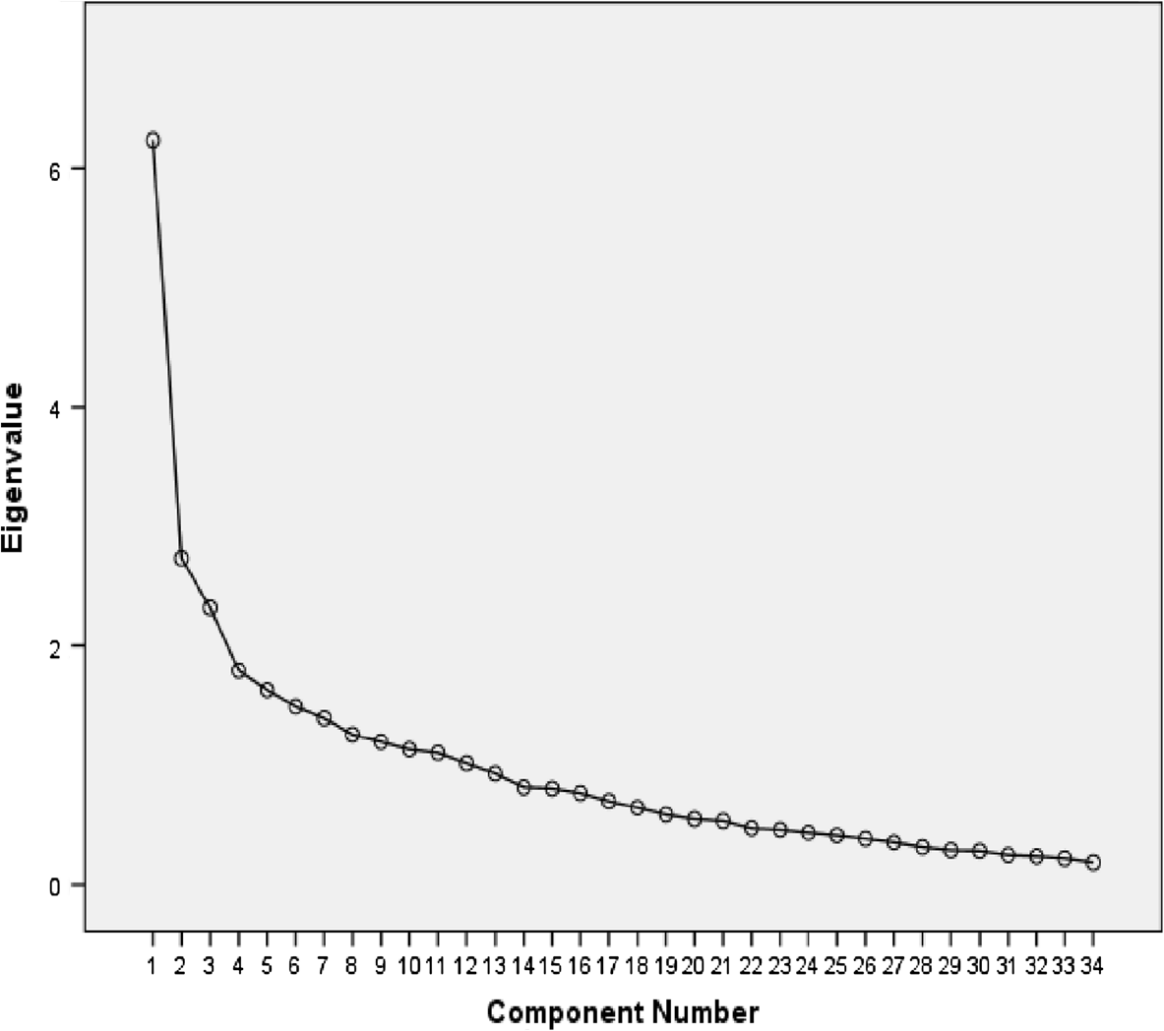
34-item MLCDP scree plot showing the amount of variance (see Appendix).

Exploratory factor analysis of the 34-item MLCDP was used to determine its construct validity [13] and to reduce the number of items if necessary [18,19] (Tables 5 and 6, Figure 1). Items that failed to load on any component or had a weak loading were removed, reducing the item number to 29 (Appendix 1).

**Table 5:** 34-item MLCDP principle component analysis describing the total variance

**Component**	**Initial Eigenvalues**			**Extraction Sums of Squared Loadings**		
	**Total**	**% of Variance**	**Cumulative %**	**Total**	**% of Variance**	**Cumulative %**
**1**	**6.23**	18.33	18.33	**6.23**	18.33	18.33
**2**	**2.73**	8.04	26.38	**2.73**	8.04	26.38
**3**	**2.32**	6.82	33.20	**2.32**	6.82	33.20
**4**	**1.79**	5.28	38.48	**1.79**	5.28	38.48
**5**	**1.63**	4.80	43.29	**1.63**	4.80	43.29
**6**	**1.49**	4.39	47.68	**1.49**	4.39	47.68
**7**	**1.39**	4.10	51.78	**1.39**	4.10	51.78
**8**	**1.25**	3.70	55.48	**1.25**	3.70	55.48
**9**	**1.19**	3.52	59.01	**1.19**	3.52	59.01
**10**	**1.13**	3.34	62.35	**1.13**	3.34	62.35
**11**	**1.10**	3.25	65.61	**1.10**	3.25	65.61
**12**	**1.02**	2.99	**68.61**	**1.02**	2.99	68.61
13	.93	2.74	71.35			
14	.81	2.40	73.76			
15	.80	2.37	76.12			
16	.76	2.25	78.38			
17	.70	2.06	80.44			
18	.64	1.90	82.35			
19	.59	1.73	84.08			
20	.55	1.62	85.71			
21	.53	1.57	87.28			
22	.47	1.39	88.68			
23	.46	1.36	90.04			
24	.43	1.28	91.33			
25	.41	1.22	92.55			
26	.38	1.13	93.69			
27	.35	1.04	94.74			
28	.31	.92	95.67			
29	.29	.85	96.52			
30	.28	.83	97.35			
31	.25	.73	98.09			
32	.23	.70	98.79			
33	.22	.65	99.44			
34	.18	.55	100.00			

Extraction Method: Principal Component Analysis.

**Table 6:** Factor analysis of the Major Life Changing Decisions Profile (MLCDP): Varimax rotated matrix with item loading (34 items)

**MLCDP Items**	**Components**			
	**1**	**2**	**3**	**4**
Seek fertility treatment	**.658**		.146	
Study near home	**.639**		.149	-.185
Move back to home area	**.603**	.196	.139	.109
Not to marry or have a long term partner	**.594**		-.168	.303
Leave college/university education early	**.534**		.292	
Move to another city	**.519**	.125	.255	-.122
Change study subject	**.514**		.341	-.228
Change plans for having children	**.504**		.175	
Leave school education early	**.496**			.147
Divorce or separation from partner	**.486**		-.134	.417
Not to buy my own home	**.430**		.107	.386
Not to have more children	.177	-	.137	.167
Involved in community activities	.147	**.800**		.147
Not to socialise	.113	**.749**		.205
Not to go swimming		**.663**	.273	
Not to take part in sports activities		**.645**	.275	.243
Wear different types/colour clothes/shoes		**.588**	.206	
Travel or holidays abroad	.156	**.578**		.143
Wear make up	.168	**.423**		
Change my eating habits		.381		.247
Change to different sports activities		.370	.270	
Select a job/career suitable for health			**.691**	
Completely change job/career	.121	.134	**.671**	.139
Change choice of job/career	.137		**.650**	.206
Give up job career after starting		.120	**.585**	.507
Flexible working hours	.155	.126	**.584**	
Shorter working hours	.177	.195	**.483**	
Not to take promotion			**.444**	.146
Move to another part of the country	.148		.275	
Take early retirement				**.700**
Remain unemployed		.141	.223	**.575**
Move my home	.198		.140	**.517**
Not to have a sexual relationship		.154		**.484**
Change my smoking/drinking/alcohol habits		.214		**.406**

Extraction Method: Principal Component Analysis. Rotation Method: Varimax with Kaiser Normalization.

**Figure 2:**
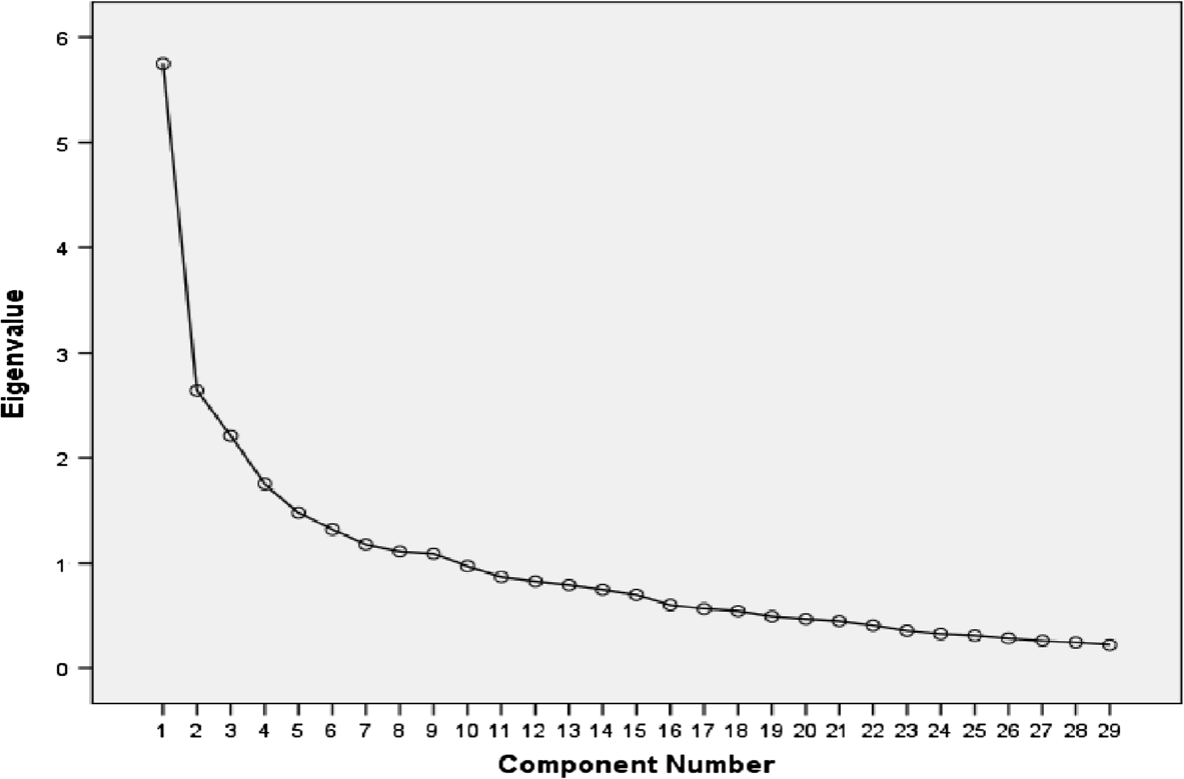
29-item MLCDP scree plot showing the amount of variance (see Appendix).

Factor analysis was carried out on this 29-item MLCDP to determine whether the remaining items fitted well under the appropriate domains (Table 7, Figure 2). This suggested that a further 6 items could be deleted, reducing the MLCDP to 23 items (Appendix 1).

**Table 7:** 29-item Major Life Changing Decisions Profile (MLCDP) varimax rotated matrix with items loading

**MLCDP Items**	**Components**		
	**1**	**2**	**3**
Not to socialise	**.693**		.215
Involved in community activities	**.683**		.201
Not to take part in sports activities	**.632**		.377
Take early retirement	**.548**	.139	-.160
Travel or holidays abroad	**.535**	.127	
Not to go swimming	**.534**		.426
Wear different types/colour clothes/shoes	**.471**		.344
Remain unemployed	**.454**	.176	.126
Not to have a sexual relationship	**.446**	.126	-.125
Change my smoking/drinking/alcohol habits	**.400**		
Move my home	.322	.272	
Wear make up	.311	.103	.201
Seek fertility treatment		**.672**	.153
Not to marry or have a long term partner	.235	**.612**	-.194
Move back to home area	.190	**.611**	.148
Study near home	-.167	**.599**	.214
Leave college/university education early		**.558**	.268
Divorce or separation from partner	.308	**.542**	-.191
Move to another city		**.510**	.280
Change study subject	-.129	**.499**	.393
Change plans for having children		**.489**	.197
Leave school education early	.151	**.467**	
Select a job/career suitable for health			**.725**
Completely change job/career	.143	.174	**.648**
Change choice of job/career	.155	.181	**.599**
Flexible working hours		.156	**.589**
Shorter working hours		.133	**.551**
Give up job career after starting	.398	.163	**.446**
Not to take promotion	.129		.387

Extraction Method: Principal Component Analysis. Rotation Method: Varimax with Kaiser Normalization.

Rotation converged in 7 iterations.

In order to ensure that all perspectives were considered in the decisions relating to retention and deletion of items, factor analysis of the full original 41-item MLCDP was also carried out. This analysis suggested that 11 items be deleted: these were compared with the 18 items suggested for deletion by the factor analysis of the 32-item MLCDP (Table 8). Nine of these were the same, supporting the strength of the initial analysis (Appendix 1).

**Table 8:** Comparison of the items deleted from the 41-item and the 32-item Major Life Changing Decisions Profile (MLCDP) after exploratory factor analysis

Comparison of two different EFA*	**Item number**	**Full description of the removed item**
Common items deleted	B9	I decided to become self-employed
	C2	I decided not to have more children
	D7	I decided to move from one country to another
	E4	I decided to be more physically active
	D6	I wanted to move abroad but decided not to
	E3	I decided to change to different sporting activities
	E5	I decided to give up driving
	D4	I decided not to buy my own home
	D14	I wanted to wear make up but decided not to
Items only deleted after factor analysis of the 32-item MLCDP along with the above 9 common items	C3	I decided not to have any children
	D15	I decided to wear a wig/toupee
	D10	I decided to move to another part of the country
	D1	I decided to change my eating habits
	D5	I decided to move my home
	D11	I decided to wear different types/colour of clothes/shoes
	E1	I decided not to go swimming
	A2	I decided to change my study subject
	B7	I decided not to take promotion
Items only deleted after factor analysis of the 41-item MLCDP along with the above 9 common items	B10	I decided to remain unemployed
	D2	I decided to change my smoking/drinking alcohol habits

* Exploratory Factor Analysis.

### Scale refinement

The prevalence of deleted items reported in the qualitative phase of the study was examined to assess the appropriateness of reinstating any removed items and to assist item merger decisions. Final decisions on item deletion, rephrasing or regrouping were made on the basis of the factor analyses, on item prevalence and on statistical, conceptual and philosophical grounds.

An item ranking table was used to compare the lowest ranked items with the items deleted as a result of factor analyses. If an item did not conceptually belong to a specific component, this also informed the decision whether or not to retain, remove or move that item to a more relevant component.

The level of disease influence on MLCDs was evaluated according to the percentage of patients who reported this influence. Items were ranked according to their prevalence (%) (Table 9). Only 16 items had a prevalence of <10%. The top three affected MLCD items reported by patients were related to life style and were placed in the social category: item D1. I decided to change my eating habits, 71.4%; item D2. I decided to change my smoking/drinking alcohol habits, 58.5%; and item D3. I decided not to travel or go for holidays abroad, 50.9%. Of the 18 items suggested for deletion as a result of the factor analysis of the 41-item MLCDP (with internal consistency reliability testing), 10 had a prevalence of >10%. Of the 11 items suggested for deletion as a result of factor analysis of the 41-item MLCDP (without internal consistency reliability testing), 6 had a prevalence of >10%: three of these items were first, second and fourth in the prevalence ranking (Table 10). This information was considered in the final selection of the MLCDP items.

**Table 9:** 41-item Major Life Changing Decisions Profile (MLCDP) prevalence of items ranked according to the percentage of patients who reported them

**Item number**	**Item full description**	**% of subjects reported**	**Item ranking**
D1	I decided to change my eating habits	71.4	1
D2	I decided to change my smoking/drinking alcohol habits	58.5	2
D3	I decided not to travel or go for holidays abroad	50.9	3
E4	I decided to be more physically active	48.5	4
E2	I decided not to take part in any sports activities	46.1	5
D12	I decided not to be involved in community activities	45.7	6
D13	I decided not to socialise	42.3	7
E1	I decided not to go swimming	40	8
B8	I decided to work shorter hours	36.1	9
D11	I decided to wear different types/colour of clothes/shoes	34.7	10
B6	I decided to select a job/career suitable for my health	30.9	11
B1	I decided to change my choice of job/career	28.5	12
E3	I decided to change to different sporting activities	27.1	13
B3	I decided to completely change my job/career	26.6	14
B4	I decided to take early retirement	24.2	15
B10	I decided to remain unemployed	23.3	16
B2	I decided to give up my job/career after starting	23.3	17
C5	I decided not to have a sexual relationship	22.3	18
D6	I wanted to move abroad but decided not to	22.3	19
B5	I decided to work flexible working hours	21.9	20
D5	I decided to move my home	19	21
C1	I decided to change my plans for when to have children	14.2	22
E5	I decided to give up driving	12.8	23
B7	I decided not to take promotion	10.4	24
C3	I decided not to have any children	10	25
**A3**	**I decided to study near home**	**9.5**	**26**
**D8**	**I wanted to move to another city but decided not to**	**9**	**27**
**C2**	**I decided not to have more children**	**8**	**28**
**C6**	**I decided not to marry or have a long term partner**	**8**	**29**
**B9**	**I decided to become self-employed**	**7.6**	**30**
**C7**	**I decided to get divorced or separate from my partner**	**7.1**	**31**
**D14**	**I wanted to wear make up but decided not to**	**7.1**	**32**
**A2**	**I decided to change my study subject**	**6.6**	**33**
**D9**	**I decided to move back to my home area**	**6.1**	**34**
**D10**	**I decided to move to another part of the country**	**6.1**	**35**
**D4**	**I decided not to buy my own home**	**6.1**	**36**
**A1**	**I decided to leave school education early**	**5.7**	**37**
**A4**	**I decided to leave college/university education early**	**5.7**	**38**
**C4**	**I decided to seek fertility treatment**	**4.2**	**39**
**D7**	**I decided to move from one country to another**	**2.8**	**40**
**D15**	**I decided to wear a wig/toupee**	**0.4**	**41**

**Table 10:** Comparison of items deleted as a result of both factor analyses with item prevalence/ranking

	Item deleted as a result of EFA*		**%**	**Items ranking**	**Full description of item**
	**1st EFA**	**2nd EFA**			
**Lowest in items prevalence ranking**	D15	-	0.4	41	I decided to wear a wig/toupee
	D10	-	6.1	35	I decided to move to another part of the country
	A2	-	6.6	33	I decided to change my study subject
	D7	D7	2.8	40	I decided to move from one country to another
	D4	D4	6.1	36	I decided not to buy my own home
	D14	D14	7.1	32	I wanted to wear make up but decided not to
	B9	B9	7.6	34	I decided to become self-employed
	C2	C2	8	28	I decided not to have more children
**Highest in items prevalence ranking**	D1	-	71.4	1	I decided to change my eating habits
	E1	-	40	8	I decided not to go swimming
	D11	-	34.7	10	I decided to wear different types/colour of clothes/shoes
	D5	-	19	21	I decided to move my home
	B7	-	10.4	24	I decided not to take promotion
	C3	-	10	25	I decided not to have any children
	-	D2	58.5	2	I decided to change my smoking/drinking alcohol habits
	-	B10	23.3	16	I decided to remain unemployed
	E4	E4	48.5	4	I decided to be more physically active
	E3	E3	27.1	13	I decided to change to different sporting activities
	D6	D6	22.3	19	I wanted to move abroad but decided not to
	E5	E5	12.8	23	I decided to give up driving

* Exploratory Factor Analysis.

### Synthesis of the factor analysis findings

**Figure 3:**
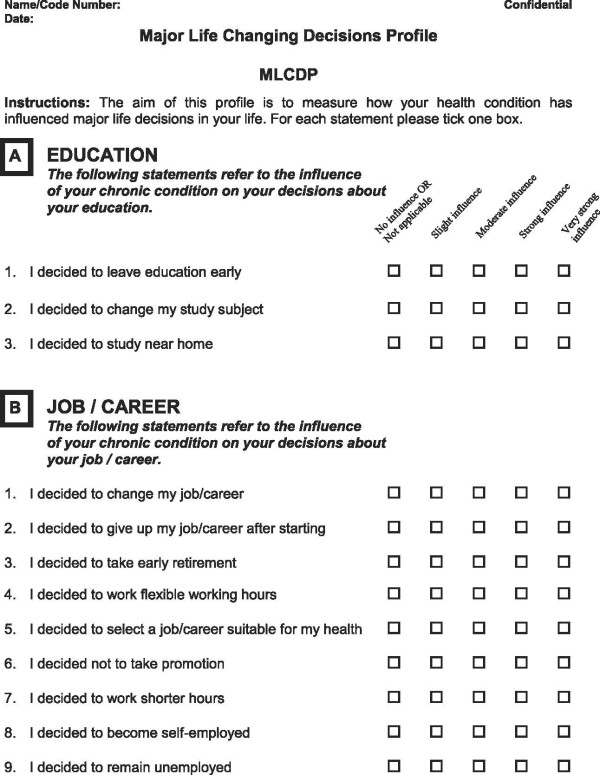
The 32-items MLCDP Part 1.

**Figure 4:**
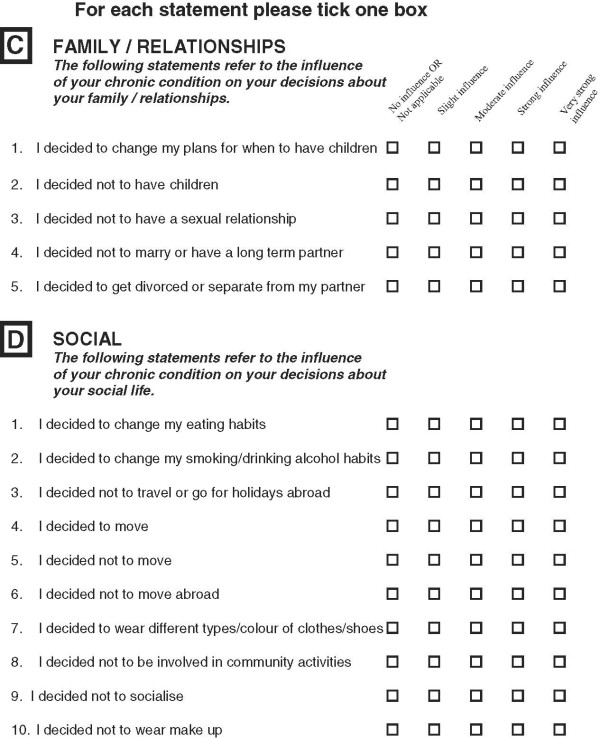
The 32-items MLCDP Part 2.

**Figure 5:**
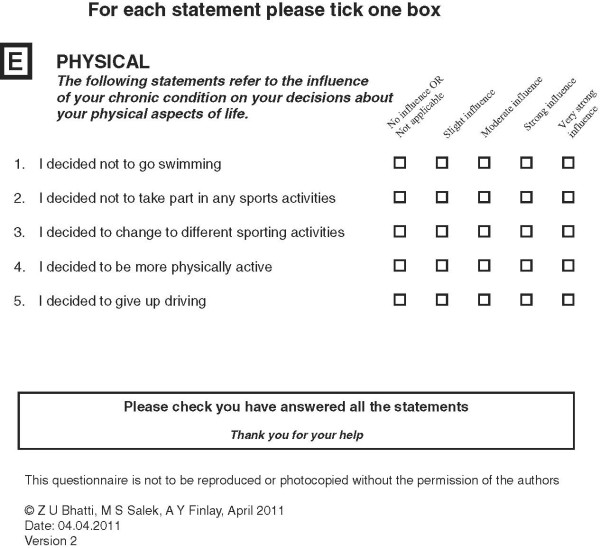
The 32-items MLCDP Part 3.

A strategy of item deletion, merger and application of appropriate phraseology was adopted for scale refinement. All 41 items were reviewed, with more attention given to those items with a prevalence of <10% and which were also suggested by one of the factor analyses to be removed (Table 10). On the basis of both factor analyses and examination of items of low and of high prevalence (items ranking), 30 items were discussed in detail. Seventeen items were retained, of which three were rephrased. Nine items were merged to create four new items and four items were completely removed. Following the scale refinement process the MLCDP items increased from 29 to 32 items. Figures 3, 4 and 5 shows the final draft of the MLCDP. The MLCDP score is expressed as the absolute number of decisions that have been affected, not as a percentage of the total number of items.

### Scoring issues

We initially considered the scoring strategy from the perspective that if a MLCD had been influenced slightly, moderately, strongly or very strongly, then different scores should be placed on the answer to that question. The scores could then be added to the various questions to give an overall score that would reflect the totality of the strength of influence of disease on scores.

As we gained more experience with the MLCDP, our approach to the philosophy of scoring has changed. Our current view is that what matters to a patient is whether or not a particular major decision has or has not been influenced by disease. Even if a decision has been influenced only slightly, it has been influenced and so the course of that patient’s life in relation to that decision has been affected by their disease. We have realised that under the graded scoring system originally proposed, if a single decision were influenced “very strongly”, this would score, say, 4, the same score as having four decisions influenced “slightly”. In reality the effect of having four decisions influenced slightly by a disease would be likely to have a much more widespread impact than having only one decision affected by a very strong influence.

Our current view is that the total number of decisions affected is the most critical information in comparing the overall impact of having a disease on MLCDs. However we currently retain the graded system of responses (without scoring them) in the MLCDP because we think that this graded information may be of value to a clinician or other carer when counselling a patient concerning this subject.

### Face validity and practicality

When the data was collected for factor analysis, all 210 patients also answered a further 6 questions to assess the face validity and practicality of the 41-item MLCDP. The mean time taken by the patients to complete the MLCDP was 5.7 minutes (range = 2–15 minutes, median = 5 minutes). 204 (97.1%) patients reported that the MLCDP was easy to complete. 198 (94.3%) patients felt that “the response options for the statements in the questionnaire” were straight forward. 131 (62.4%) patients reported that the instructions and statements were “very clear” and 76 (36.2%) patients reported them as being “clear”. 181 (86%) patients considered that the MLCDP was sufficiently comprehensive to measure the influence of disease on important life decisions. No new questions were suggested.

## Discussion

We describe above the development and initial psychometric properties of a new tool designed to measure the novel domain of the impact of disease on a patient’s MLCDs. The MLCDP was directly based on the experiences of patients.

The MLCDP is the first instrument specifically developed to measure the influence of chronic diseases on MLCDs. A strict methodology [10] was followed at all developmental stages. The MLCDP is a generic questionnaire designed to be used across all medical specialities. An adequate number of subjects (n = 210) from seven medical specialities took part in the evaluation of its initial psychometric properties. A thorough process of multiple factor analyses was carried out. The MLCDP is easy to understand and can be completed in less than six minutes.

At the initial development stage a maximum possible number of items (48) were generated from patients’ responses and grouped into six broad MLCD domains to make sure no aspect of MLCDs was overlooked. In the content validation of the MLCDP both quantitative and qualitative assessment techniques were applied [15].

“Exploratory factor analysis is a complex procedure, exacerbated by the lack of inferential statistics and the imperfections of “real world data” [20]. The researchers’ judgment concerning the deletion and retention of specific items is important and should be based on the patients’ responses and the research concept [20]. Indeed, the purpose of factor analysis was to see to what degree the mathematical approach confirms clinical intuition. It was therefore decided to keep in conceptually sound items at this stage of the instrument development, even though they may have been suggested by the factor analyses to be removed. It seemed appropriate to preserve those items for when other techniques, such as Rasch analysis and item response theory, could be applied to the data for further scale refinement.

At content validation stage we discussed with the panel of judges whether it was appropriate to include MLCDs that were confined to one particular gender or disease. This panel had decided to exclude any MLCD category that was reported by <5% of the patients. However the panel retained some gender and speciality specific categories to allow them to be tested for appropriateness and relevance at a later stage. The “make up” item came lowest in the item prevalence ranking and was also suggested by both factor analyses to be removed. But a closer consideration of this item and of related comments from patients at the scale refinement stage gave us further insight into the importance of this MLCD. The panel discussed in great detail the conundrum: does it really make sense to remove an item, if it genuinely represents from the patient’s perspective an important MLCD? It was decided to retain the “make up” item at this stage and to postpone the decision as to whether or not to retain this item until after Rasch analysis and Item Response Theory are applied to MLCDP data for further scale refinement.

All the items came directly from patients and this patient origin had the paramount influence in our decisions concerning the development of the scale. Another reason to maintain items was to create a sense of concordance with the respondent by ensuring that items would be perceived as relevant. All items were subjected to a “factor extraction” process. Factor analysis should ideally be based on 300 cases, however 150 cases should be sufficient [18] and so the sample size of 210 was considered adequate.

When deciding scoring methods and their interpretation fundamental questions were raised such as what would be the perceived “value” of a patient reporting only one MLCD influenced by their chronic condition, if viewed within the perspective of a score system based on a maximum score of 32, should such a scoring system be devised. Does the MLCDP conceptually require any scoring system? What scoring system would assist meaningful interpretation of the data? Two patients reported that their disease had influenced >10 MLCDs, indicating an extraordinary level of long-term impact on those patients’ lives. When we consider a patient in whom only one MLCD had been influenced, in the context of the 32 items identified, it might appear that the patient has experienced minimal impact on their life, but even one decision recorded as being influenced was a major decision which was life changing. There is virtually no possibility that all 32 MLCDs would be affected in any one patient and <1% patients reported more than 10 MLCDs affected. The profile is therefore more appropriately scored as an absolute value rather than as a percentage. Therefore the fundamental unit for expressing data from the MLCDP is the “total number of MLCDs that have been affected”. Further psychometric analysis will determine whether for the final scoring model a single overall score, or a series of sub-domain scores, is most appropriate. It remains to be determined whether individual item weighting should be applied.

The MLCDP is not designed to detect changes over time or to be used in follow up studies, such as before and after intervention. It may help to identify which diseases have the biggest impacts on patients’ lives; a total score based on adjectival data would be difficult to interpret in this context. However, the adjectival scoring was used for carrying out factor analysis as part of the development (item reduction) of the MLCDP in order to confirm grouping of the items based on clinical intuition through mathematical modelling. Therefore, the guidance provided for interpretation of the scores from the adjectival system has taken into account the reality of how the impact of a chronic disease on MLCDs should be managed.

Factor analysis indicated that some conceptually important items, which were high in the items prevalence ranking, should be deleted. However we decided that there should be a conservative approach to deleting items at the scale refinement stage and that decisions should be based primarily on patients’ experiences, as shared during the detailed interviews and postal survey responses. It was reassuring that nine items were suggested for removal by two different factor analyses but it could be argued that all 41 of the original items are valid items, as they were all suggested by at least one actual patient and therefore reflect patients’ reality. The tension between the outcome of factor analysis and patients’ qualitative reports of their experiences is a common phenomenon in instrumental development. It is left to the research team to resolve this tension through careful review of both sources.

### Uses of the MLCDP

The MLCDP may be helpful to identify which diseases have the biggest impact on patients’ lives in terms of their MLCDs and which disease impacts most on specific decisions. The MLCDP could be used in epidemiological studies as a measure of disease burden. The use of the MLCDP has the potential to compare the influence of different diseases or diseases of different specialties on MLCDs^3-7^. In the clinic, the MLCDP may provide clinicians with a one-off insight into how patients’ disease has already impacted their life decisions. It could be used alongside a QoL questionnaire designed to measure current impact to obtain a more complete picture of disease impact. The MLCDP could be used in long term cohort studies to identify how affected MLCDs accumulate over time.

## Conclusions

This study provides a method to evaluate a critical aspect of the long-term impact of chronic disease on patients’ lives, the influence on MLCDs, allowing the use of the “MLCD” domain in the assessment of long-term disease impact and the overall assessment of HRQoL.

The MLCDP data could be used to inform health, education and employment policies underpinned by a patient-centred approach. The use of the MLCDP could help with educating health providers about long-term consequences of chronic conditions, preparing them to help patients at an early disease stage and offer a long-term management plan. The use of the MLCDP for epidemiological surveys could identify which chronic conditions have the most influence on which type of MLCDs.

### Limitations and further work

Further psychometric testing is required. As some items were deleted, merged or rephrased, further factor analysis and internal consistency reliability of the remaining items is required, followed by internal consistency reliability and test-retest reliability of the final remaining items. Full psychometric evaluation of the MLCDP should be carried out using both the classical test theory approach and novel techniques, such as item response theory. The MLCDP was not designed to detect change, but to be used to identify the number of major life decisions that a patient considers have been affected by their disease. We would therefore not expect there to be differences in the “behaviour” of the instrument according to different duration of disease, but this will need to be tested.

The current profile is not designed to detect change over time or to be used in follow up studies, such as before and after drug intervention. Further work is required to create a tool similar to the MLCDP that would be sensitive to clinical change. The issues surrounding MLCDs are very complex and highly subjective. There is therefore a need to study a control group to see whether there is a difference in approach between people who have or who do not have chronic disease and to confirm our assumption that MLCDs are part of the normal course of life. The MLCDP is an easy to complete generic tool. Although it requires further validation, it provides a means to measure this major impact of disease on patients’ lives.

### Ethical approval

The study was approved by the South East Wales Local Research Ethics committee (2nd June 2008). All participants consented to take part before starting the study.

## Appendix 1

Factor analysis details

Reliability of the 41-item MLCDP

The Cronbach’s alpha reliability of the 41-item MLCDP was 0.84, indicating good reliability. The “corrected items-total correlation” values for 7 items were <0.2 (not discriminating well) and therefore deleted, changing Cronbach’s alpha from 0.84 to 0.85. The 7 deleted items were B9 “I decided to become self-employed”, C3 “I decided not to have any children”, D6 “I wanted to move abroad but decided not to”, D7 “I decided to move from one country to another”, D15 “I decided to wear a wig/toupee”, E4 “I decided to be more physically active” and E5 “I decided to give up driving”.

Factor Analysis of 34-item MLCDP

Exploratory factor analysis of the remaining 34 items of the MLCDP was carried out on the sample of 210 [13]. In the correlation matrix for the 34 items of the MLCDP there were correlation coefficients r = 0.3 and above [18] between several variables, confirming the suitability of the data for carrying out factor analysis. The Kaiser-Meyer-Olkin (KMO) [21,22] value was 0.73, greater than the recommended minimum of 0.6 [18] and Bartlett’s test of sphericity [23] was significant at p = 0.0001 confirming the sampling adequacy of the data. Kaiser’s criterion and Cattell’s scree test statistical techniques were used to determine how many factors could be extracted.

There were 12 components with Eigenvalues >1, that were retained for further analysis [18]. These 12 components explained 68.6% of the variance (Table 5) [20]. The component matrix for the 34 items MLCDP showed the loadings of 34 items on 12 components. Most of the items loaded strongly (0.3 and above) on the first six components and these items were considered for future analysis. 20 items had values of >0.40.

In the scree plot [24] (Figure 1), four components were retained for further analysis. There is a sharp drop after the first factor indicating that the first factor accounts for most of the variance (10.5%). Using the factor rotation technique, the varimax rotated component matrix [18] revealed that after rotation, thirty items loaded strongly (>0.40) (Table 6) on one of the four components, indicating a strong correlation between the item and the corresponding components. Most items loaded on component 1 (11), component 2 (7) and component 3 (7). Only two items failed to load on any component, and were therefore deleted.

The four factors identified from the rotation accounted for 38.4% of the total variance, along with their percentage of variance explained. The first four factors accounted for 10.5%, 10.2%, 9.8% and 7.8% of the total variance explained. After rotation, the pattern of the percentage of variance of individual components and their cumulative percentage changed from the total variance explained earlier. However, cumulative total variance explained (38.4%) did not change after rotation.

To summarise this first factor analysis, in the rotated component matrix, two items failed to load into any component. There were two items with weak loading (<0.4) and one item loaded on multiple components with no significant difference in their values. These five items were removed, leaving a 29-item MLCDP.

Factor Analysis of 29-item MLCDP

Factor analysis of the 29-item MLCDP was carried out to see whether these remaining items fitted well together in their components. From the scree plot examination (Figure 2) three factors were extracted for further analysis. This scree plot was relatively improved compared to the previous scree plot where four factors were extracted. Varimax rotation was applied, confirming the initial structure of the scale (Table 7). All 29 items loaded to 3 extracted components. 26 items loaded highly (0.4 and above), 3 items loaded weakly (<0.4). Six items loaded on two components, and for 3 of these, the values on two components were very close (weak complex variables). Five or more strongly loading items (0.5 or above) are desirable to create a solid factor [20]. Component 1 (factor) consisted of 12 items of which only 2 items loaded weakly (range = 0.31 to 0.69). Component 2 consisted of 10 items with factor loading ranging from 0.46 to 0.67, with no weak loading. Component 3 consisted of 7 items with loadings ranging from 0.38 to 0.72, with only one weak loading. Total variance explained of the extracted component, demonstrated that the 3 factors accounted for 36.5% of the total variance. Although there were 5 more items in the first rotation and four components were extracted, there was not much difference compared to the percentage of total variance explained after the first rotation (38.4%). The first factor accounted for the highest proportion of variance, 12.7%. The second and third factors accounted for a similar proportion of the variance, 11.9% and 11.8% respectively.

The majority of items were close to each other in their corresponding components. For example, component 1 consisted of 12 items of which 9 items dealt with MLCDs related to social and physical aspects of patients’ lives. Six of the 29 items that weakly loaded or had complex variables were considered for deletion, leaving a 23-item MLCDP. Therefore as a result of factor analysis 18 MLCDP items were suggested for deletion from the original 41-item MLCDP, but were considered again at the next refinement stage of the scale. Items which did not conceptually fit in their extracted corresponding factors were also discussed at this stage.

In order to ensure that all perspectives were considered in the decisions relating to retention and deletion of items, factor analysis of the full original 41-item MLCDP was also carried out. Items were deleted after the final varimax rotation based on the same criteria used for the previous analysis. The final rotated component matrix revealed that four items failed to load on any component, five items loaded weakly (<0.4) and two items loaded on two components with little difference between their values. These 11 deleted items were compared with the 18 items deleted as a result of the factor analysis of the 32-item MLCDP (Table 8). Nine of the items were the same, which supported the initial factor analysis approach.

## Abbreviations

MLCDs: Major life changing decisions; MLCD: Major life changing decision; MLCDP: Major life changing decision profile; HRQoL: Health-related quality of life; SPSS16: Statistical package for the social sciences; KMO: Kaiser-Meyer-Olkin; QoL: Quality of life

## Competing interests

The authors declare that they have no competing interests.

## Authors’ contributions

ZUB conducted the study, collected and analysed the data. ZUB and AYF wrote the initial draft of the manuscript. MSS and AYF directed and supervised the study and redrafted and produced the final version of the manuscript. All other authors contributed to the clinical study and read, contributed to and approved the final manuscript.
